# Social Isolation and Loneliness: The Pros and Cons of Technological Interventions

**DOI:** 10.1093/geroni/igaa057.2515

**Published:** 2020-12-16

**Authors:** George Demiris

**Affiliations:** University of Pennsylvania, Philadelphia, Pennsylvania, United States

## Abstract

A variety of technologies have been proposed, tested, and used to reduce social isolation and loneliness [SIL]. Furthermore, information technology may offer a way to detect or predict patterns of SIL. In this paper, the range of technology tools for assessment (e.g., passive monitoring, tracking data patterns, electronic health records) and intervention (e.g., social robots, social media, virtual reality) are reviewed for evidence of impact, and consideration is given for related ethical issues. The role of technology in relation to the report’s recommendations is discussed, including assessment and testing of new technological interventions for their potential benefits and harms, consideration of contextual issues such as broadband access, and the role of technology in education and training. Finally, gap areas of research are explored, such as the impact of the use of technology among current younger adults as they age. Part of a symposium sponsored by Loneliness and Social Isolation Interest Group.

